# Torsion of a giant pedunculated liver hemangioma mimicking acute appendicitis: a case report

**DOI:** 10.1186/1749-7922-5-2

**Published:** 2010-01-18

**Authors:** Feyzullah Ersoz, Ozhan Ozcan, Ahmet Burak Toros, Serdar Culcu, Hasan Bektas, Serkan Sari, Esra Pasaoglu, Soykan Arikan

**Affiliations:** 1 Istanbul Education and Research Hospital, Department of General Surgery, Istanbul, Turkey; 2 Istanbul Education and Research Hospital, Department of Gastroenterology, Istanbul, Turkey; 3 Istanbul Education and Research Hospital, Department of Pathology, Istanbul, Turkey

## Abstract

Hemangiomas are the most common benign neoplasms affecting the liver. They occur at all ages. Most cases are asymptomatic and do not require any treatment. Rarely, hemangiomas can be pedunculated. İf they undergo torsion and infarction, they become symptomatic. Herein; we report the case of a 31 year old male presenting with features of acute appendicitis: continuous right iliac fossa pain, rebound, guarding tenderness at McBurney' s point, nausea, anorexia, shifted white blood cell count and a Mantrels score of 6. At laparotomy a normal appendix was observed and a torsioned pedinculated liver hemangioma turned out to be the cause.

## İntroduction

Hemangiomas are the most common benign neoplasms affecting the liver with an incidence of 0.4-20% in autopsy series [1]. Women are affected more often than men. The female-to-male ratio is 5:1 to 6:1. They occur at all ages. Most cases are asymptomatic and do not require any treatment. Pedunculated haemangiomas are extremely rare, with only a few cases reported in the literature [2].

Herein; we report the case of a torsioned giant pedunculated liver haemangioma that mimicked acute appendicitis.

## Case Presentation

**Figure 1:**
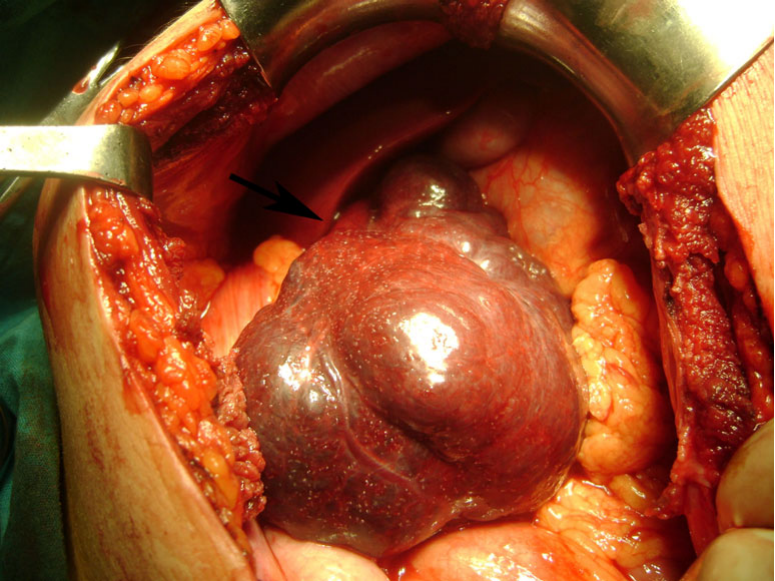
**Pedinculated hemangioma on the operation table; black arrow points the pedincule**.

**Figure 2:**
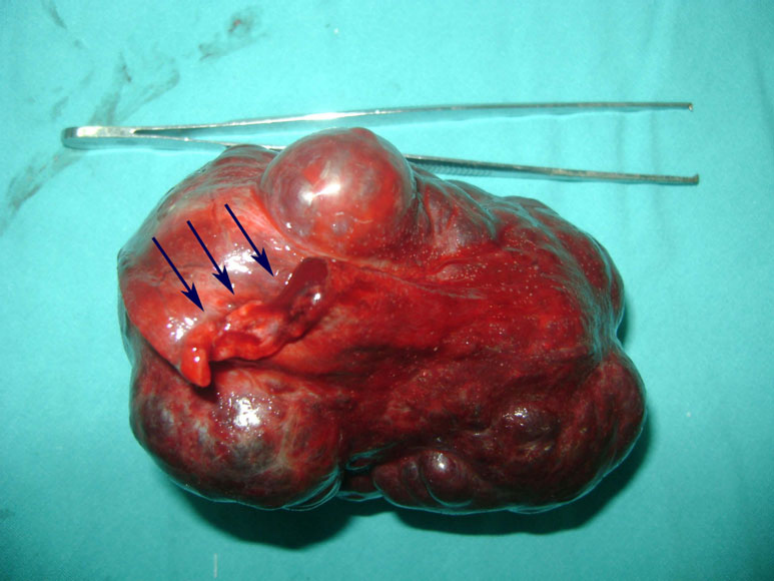
**Resected hemangioma; arrows point the pedincule**.

**Figure 3:**
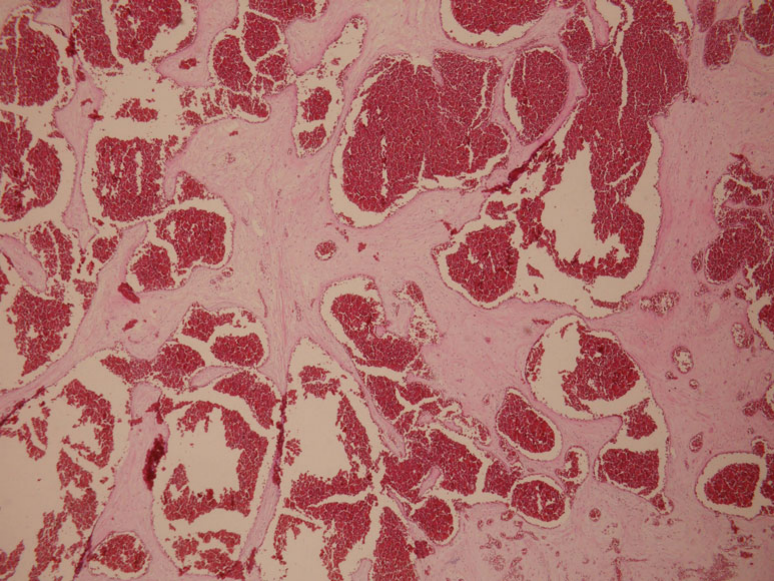
**Histopathologically the lesion composed of large vessels with cystically dilated lumina and thin walls**. Lumen of blood vessels is filled with erythrocytes.(H+E).

A 31 year old man admitted to our emergency department with a 2 day history of right iliac fossa pain which he described as continuous. He also had anorexia, nausea. On physical examination, his pulse rate was 96 beats/min, his body temperature was 37.1°C. His abdomen was markedly tender at the right iliac fossa with guarding and rebound tenderness at McBurney's point. The rest of the systemic examination was normal and the Mantrels score of the patient was 6. Laboratory data was as follows; hemoglobin 15.8 g/dl, total leukocyte count 9700/mm3, with 75% polymorphonuclear leukocytes, 37% lymphocytes, 3,2% monocytes, and 1% eosinophils; erythrocyte sedimentation rate was 2 mm for 1 h. Liver function tests, serum electrolytes, and creatinine were all within normal ranges. His bowel movements were regular on oscultation. Per rectum examination was normal. The abdominal X-ray was normal and because of the manifest clinical picture, abdominal ultrasound was not performed. Under the light of medical history and signs on abdominal examination, the patient was diagnosed as having acute appendicitis with a Mantrels score of 6 and was taken to theatre for appendectomy. At operation a normal appendix was found. At further exploration, a large soft reddish mass was palpated near the caecum. Macroscopically, the mass measured 10 × 12 × 15 cm. It was connected to the right inferior margin of the liver with a thin pedincule. It had undergone a 360° clockwise torsion on its pedincule. The mass was easily detorsioned and resected (Fig 1 and 2). Appendectomy was also performed using the routine method. Histologic assessment confirmed a cavernous hemangioma. The mass had multiple vascular spaces and fibrosis and was unusual for that there was a considerable amount of adipocytes intermingling within the tumor (Fig 3). The patient's recovery was uneventful, and he was discharged on the 2nd postoperative day.

## Discussion

Cavernous hemangioma is the most common benign tumor of the liver. They are probably of congenital origin and have no potential for malignant transformation. Most are diagnosed incidentally and are asymptomatic. Hemangiomas are usually found at the right lobe of the liver in a subcapsular or marginal location. Most hemangiomas are diagnosed incidentally and are small and asymptomatic. Their size usually remains stable and can vary from a few milimetres to more than 20 cm. Lesions larger than 4 cm have been defined as giant hemangiomas [3].

Giant hemangiomas may cause abdominal discomfort, swelling, abdominal pain, icterus and thrombocytopenia [4]. Very rarely, spontaneous rupture with intraabdominal hemorrhage may create acute abdominal symptoms, which may also occur after rupture due to blunt abdominal trauma. Surgery is the treatment of choice, especially for giant, symptomatic hemangiomas with uncertain diagnosis.

Rarely, hemangiomas can be pedunculated [5]. At ultrasound, the origin of the lesion may be difficult to recognize. The lesion can be attached to the liver with a thin pedicle, which is nearly undetectable at imaging. If they undergo torsion due to their long, mobile pedincule and get infarcted, they may become symptomatic. Pain is the most frequent symptom and most likely occurs from infarction or pressure on surrounding tissues. They can seldom cause pressure symptoms or get ruptured. Definite diagnosis should be made to distinguish it from other causes of acute abdominal pain.

To the best of our knowledge, this present case is the first example of a torsioned pedunculated hemangioma in the literature, mimicking acute appendicitis with a Mantrels score of 6 [6].

In conclusion, this case report impresses that; even incidentally detected pedunculated hemangiomas must be managed by surgery for their tendency to get torsioned. In addition; the surgeon must look for different ethiologies when a normal appendix is found during operation.

## Competing interests

The authors declare that they have no competing interests.

## Authors' contributions

All the authors participated in the admission and the care of this patient, the conception, the design, data collection and interpretation, manuscript preparation and literature search.

All authors have read and approved the final manuscript

## Consent

Written informed consent was obtained from the patient for publication of this case report and any accompanying images. A copy of the written consent is available for review by the Editor-in-Chief of this journal.
